# The role of serum uric acid in the prediction of graft‐versus‐host disease in allogeneic hematopoietic stem cell transplantation

**DOI:** 10.1002/jcla.23271

**Published:** 2020-03-02

**Authors:** Katayoon Ghasemi, Sayeh Parkhideh, Mohammad Hossein Kazemi, Maryam Salimi, Sina Salari, Ronak Nalini, Abbas Hajifathali

**Affiliations:** ^1^ Hematopoietic Stem Cell Research Center Shahid Beheshti University of Medical Sciences Tehran Iran

**Keywords:** Allo‐HSCT, GVHD prediction, uric acid

## Abstract

**Background:**

Uric acid (UA) level is of the valuable signs of inflammation. However, the role of UA in the outcomes of hematopoietic stem cell transplantation (HSCT) such as GVHD and patients’ overall survival is still a matter of debate. In this study, we aimed to evaluate the relationship between UA levels and GVHD incidence and overall survival in allogeneic HSCT patients.

**Methods:**

A total of 201 patients who were admitted for allogeneic transplantation at Taleghani hospital, Tehran, Iran, were considered for retrospective analysis. Serum UA levels from 1 week before transplantation until 2 weeks after transplantation were used to determine thresholds and find out the association of serum UA levels with GVHD and overall survival.

**Results:**

We showed that the determined thresholds using receiver operating characteristic curves have poor predictive value for GVHD and overall survival. The patients with serum UA higher than 3.4 mg/dL had 37% lower odds of GVHD incidence and 35% lower hazard of death than patients with UA lower than 3.4 mg/dL.

**Conclusion:**

Our results indicated that serum UA levels lower than 3.4 mg/dL could significantly increase the incidence of GVHD and hazard of death. The antioxidant functions of UA could explain the lower incidence of GVHD in hyperuricemic patients. However, the inconsistencies of the previous studies require further investigation to elucidate the role of UA in the prediction of GVHD.

## 1 INTRODUCTION

Today, the rate of allogeneic hematopoietic stem cell transplantation (allo‐HSCT) continues to increase. About 50 000‐60 000 transplantations are performed annually, worldwide.^1^ Graft‐versus‐host disease (GVHD) is a major complication and therapeutic challenge of allo‐HSCT with the prevalence of 20%‐60%.^2^, ^3^ During the progression of GVHD, donor driven T cells can be primed against recipient antigens and cause severe morbidity and mortality. There are two types of GVHD, including acute (aGVHD) and chronic (cGVHD).^4^ The median time of aGVHD and cGVHD is typically 21‐25 days and 4.5 months, respectively, with the incidence of 30%‐50% and 30%‐70%, in the order given.^5^ There are many risk factors affecting the development of GVHD in HSCT patients, including type of the graft, donor‐recipient relationship, HLA and sex matching, recipient age, intensity of conditioning and prophylaxis regimens.^6^ The previous studies identified that HSCT process alters serum uric acid (UA) levels in allo‐HSCT.^7^ During HSCT process, conditioning regimen leads to promotion of endogenous danger signaling such as heat shock proteins and UA.^8^ Moreover, radio/chemotherapy induces UA crystallization and deposition by increasing serum UA which can result in neutrophil migration into tissues and intensifying the inflammation.^9^ UA from damaged cells releases into extracellular fluid and induces antigen‐presenting cells (APCs) for activating the immune response.^10^ In fact, UA is a danger‐associated molecular pattern (DAMP) capable of inducing T cells to release IL‐1B through the activation of the NOD‐like receptor protein (NLRP) 3.^11^ Regarding the previous studies indicating the UA‐mediated activation of host APC and T‐cell response, the elevated UA levels may play a critical role in the immune activation and inflammation.^10^, ^11^ There are several studies about the relationship between UA levels and the incidence of GVHD and overall survival in allo‐HSCT patients with controversial findings.^12^, ^13^, ^14^, ^15^ Formerly, UA‐decreasing drugs such as uricase or allopurinol in allo‐HSCT patients have been shown to inhibit cytotoxic T lymphocytes activity and reduce the development of aGVHD.^12^ On the other hand, a recent study found a negative association between UA levels and GVHD showing the need for more clarification.^14^ Therefore, in this study, we aimed to evaluate the relationship between UA levels as a sensitive parameter and GVHD incidence as well as overall survival in patients who underwent allo‐HSCT.

## 2 METHODS AND PATIENTS

### 2.1 Patient's characteristics

A total of 201 patients with hematological disorders who were admitted for allogeneic transplantation at Taleghani hospital, Tehran, Iran, between 2008 and 2018 were considered for retrospective data analysis. All patients were categorized based on their diagnosed disease, including acute myeloid leukemia (AML), acute lymphoid leukemia (ALL), aplastic and Fanconi anemia (AA, AF), Hodgkin disease (HD), non‐Hodgkin lymphoma (NHL), and others. These patients gave the informed consent that their data were used for scientific analysis, and the study was approved by the Ethics Committee of the Shahid Beheshti University of Medical Sciences (Tehran, Iran). The samples of bone marrow were used for disease diagnosis by standard techniques such as karyotyping, hemograms, and cell surface marker detection. High‐dose chemotherapy‐based myeloablative conditioning (MAC) regimens were administered for allo‐HSCT including ALL, AML, and CML received MAC‐1 regimen (busulfan [BU; Otuska]: 3.2 mg/kg/d from day −7 to −4 and cyclophosphamide [CY; Sandoz]: 60 mg/kg/d on day −3 and −2) or MAC‐2 (BU 3.2 mg/kg/d from day −6 to −3 and fludarabine [Flu; Genzyme] 30 mg/m^2^ of body area/d from day −6 to −3) or MAC‐3 which is MAC‐2 plus antithymocyte globulin (ATG; Genzyme) (1.5 mg/kg/d on days −3, −2, and −1). Aplastic and Fanconi anemia patients received CY 60 mg/kg/d on day −3 and −2 with ATG (1.5 mg/kg/d on days −3, −2, and −1). Patients with HD and NHL received Reduced Intensity Conditioning regimen (RIC) consists of Flu 30 mg/m^2^ of body area for 5 days, CCNU (Lomustine, Bristol Myers) 100 mg/m^2^ for 2 days and Melphalan (Alkeran; GlaxosmithKline) 40 mg/m^2^ for 1 day. GVHD prophylaxis was methotrexate (MTX; Sandoz) plus cyclosporine A (CSA; Sandoz) with or without ATG. The course of methotrexate therapy was 10 mg/m^2^ of body surface on days +1, +3, +6, and +11 and for cyclosporine was 5 mg/kg/d in two divided doses on day −5. The level of cyclosporine was maintained between 200 and 300 ng/mL. Also, the ATG was given as 1.5 mg/kg/d on days −3, −2, and −1.

### 2.2 HSCT process and GVHD evaluation

The peripheral blood stem cells (PBSC) were mobilized after 4 days of 10 mg/kg G‐CSF (filgrastim; Amgen) administered in HSCT donor. Apheresis time was 250 minutes depending on the volume and speed of blood flow of PBSC donor. Cell viability in apheresis product was assessed using the trypan blue staining (Sigma‐Aldrich; Merck Millipore) and enumeration on a Neubauer chamber with light microscopy. Also, the counts of CD34+ (PE‐conjugated, EXBIO) cells and CD3+ (FITC‐conjugated; Beckman Coulter) cells were evaluated by flow cytometry (Attune NxT; Life Technologies).

The standard clinical signs such as rash, diarrhea, and liver function abnormalities associated with biopsy and histopathological criteria in involved organ were main manifestations for diagnosis of GVHD according to the GVHD criteria provided by National Institute of Health.^16^

### 2.3 Laboratory test

Serum UA levels were measured from day −7 until day +14. Peripheral blood samples were collected, and UA levels in serum samples were evaluated using an ADVIA 1800 clinical chemistry analyzer (Toshiba).

### 2.4 Statistical analysis

Data were expressed as means ± standard deviation (SD) or frequency (%). The main purpose of this study was to identify the effects of serum UA on the incidence of GVHD and the overall survival with the adjusted effect of other risk factors. The logistic regression was employed when the outcome variable was GVHD. The odds ratios, 75 and 90% confidence interval (CI), were calculated accordingly. When the outcome was the overall survival, we conducted a Cox regression analysis. The hazard ratios, 75 and the 90% CI were evaluated as well. The median of serum UA in each disease category as well as the correlation of UA level with the overall survival was determined.

The threshold for serum UA level in three different periods of time including 1 week before transplant, 1 and 2 weeks after transplant was determined using both the receiver operating characteristic (ROC) curves and median. The sensitivity, specificity, and the area under curve (AUCs) were also assessed in ROC curves analysis. Finally, due to the importance of the week before transplantation, the median of UA level in the time period between day −7 to the transplantation day was selected as the cutoff and utilized in the univariable and multivariable analysis. The survival curve was drawn based on the median of serum UA. The *P*‐value <.25 in univariable analysis and *P*‐value <.1 in multivariable was considered as significant. All analyses were conducted through the SPSS version 19.0 (SPSS Inc).

## 3 RESULTS

### 3.1 Descriptive statistics and UA cutoff determination

The descriptive statistics is shown in Table 1. The analysis was performed on 201 patients with hematological disorders with a mean age of 32.53 years. The most diagnosed disease was AML comprising 48.8% of all patients. About half of the patients 103 (51.2%) received MAC1 and approximately more than 74% of the recorded conditioning regimens were MAC1 and MAC2. The median of serum UA levels in the period of 1 week before the transplantation was 3.4 mg/dL which is selected as cutoff for the statistical analysis. Almost 45% of the patients had UA levels lower than 3.4 mg/dL.

**Table 1:** Descriptive statistics

Variables	Frequency (Mean ± SD)
Recipient age	32.53 ± 10.83
Missing	8 (4)
Donor age	31.81 ± 11.26
Missing	64 (31.8)
DP gender	
M‐M	56 (27.9)
M‐F	59 (29.4)
F‐F	31 (15.4)
F‐M	48 (23.9)
Missing	7 (3.5)
Diagnosed disease	
NHL	13 (6.5)
HD	12 (6.0)
AML	98 (48.8)
ALL	51 (25.4)
AA	8 (4)
Other	6 (3)
Missing	13 (6.5)
Recipient CMV IgG	
Positive	84 (41.8)
Negative	12 (6.0)
Missing	105 (52.2)
Conditioning regimen	
MAC1	103 (51.2)
MAC2	46 (22.9)
MAC3	16 (8.0)
RIC	21 (10.4)
AA‐AF	7 (3.5)
Missing	8 (4.0)
Prophylaxis regimen	
CSA + MTX	124 (61.7)
CSA + MTX+ATG	20 (10)
Unclassified	57 (28.4)
Uric acid	
Higher than 3.4	85 (42.3)
Lower than 3.4	90 (44.8)
Missing	26 (12.9)

### 3.2 Predictive values of the serum uric acid level before and after HSCT for GVHD

Beside the median of serum UA levels during the week before transplantation as the main cutoff point for UA in this study, the ROC curve was also drawn to determine a cutoff and evaluate the predictive values of serum UA level before and after transplant for GVHD. As shown in Table 2, the AUC, sensitivity, and specificity for the meantime of 1 week before transplant for GVHD were 59% (CI: 0.50‐0.67), 76%, and 39%, respectively with a cutoff value of 2.98 mg/dL. Therefore, it can be suggested that serum UA level of 7 days before transplant can be used as a relatively poor predictive of GVHD since the AUC is close to 60%. The cutoff value of UA level in 1 week after transplantation for GVHD was 3.91 mg/dL with the AUC of 47% (CI: 0.37‐0.56), sensitivity of 29%, and specificity of 82%. So, it is suggested that the serum UA level for the meantime of the 7 days after transplant cannot be a predictive of GVHD, since the AUC is below 50%. For the meantime of 2 weeks after transplant for GVHD, the AUC, sensitivity, and specificity were 54% (CI: 0.44‐0.63), 45%, and 67%, one by one with a cutoff value of 4.23 mg/dL. Thus, we can say that the serum UA level for the meantime of 2 weeks after transplant may be a poor predictive of GVHD, since the AUC is close to 60.

**Table 2:** Optimal thresholds for uric acid in different periods of time for predicting GVHD

Period of time	Thresholds	Sensitivity (%)	Specificity (%)	AUC(CI)
(−7 to 0)	2.98	76	39	59% (50‐67)
(0‐7)	3.91	29	82	47% (37‐56)
(7‐14)	4.23	45	67	54% (44‐63)

### 3.3 Predictive values of the serum uric acid level before and after HSCT for overall survival

In order to determine the predictive values of serum UA level before and after transplant for overall survival, we performed ROC analyses. The results are indicated in Table 3. The AUC, sensitivity, and specificity for the meantime of 1 week before transplant for OS were 53% (CI: 0.42‐0.64), 45%, and 69%, respectively, with cutoff value of 3.79 mg/dL. For the meantime of 1 week post‐transplant, the AUC, sensitivity, and specificity were 50% (CI: 0.39‐0.62), 37%, and 89%, successively with a cutoff value of 4.10 mg/dL. The cutoff value of serum UA level for 2 weeks post‐transplant was 4.66 mg/dL with the AUC of 53% (CI: 0.42‐0.64), sensitivity of 42%, and specificity of 76%. All of these AUCs were around 50%. Hence, none of them can be strong predictive of OS.

**Table 3:** Optimal thresholds for uric acid in different periods of time for predicting of OS

Period of time	Thresholds	Sensitivity (%)	Specificity (%)	AUC(CI)
(−7 to 0)	3.79	45	69	53% (42‐64)
(0‐7)	4.10	37	89	50% (39‐62)
(7‐14)	4.66	42	76	53% (42‐64)

### 3.4 Association of high serum UA and other risk factors with GVHD

We conducted a univariable and multivariable logistic regression to identify the pre‐transplant risk factors for GVHD. The results are illustrated in Table 4. As we can see in the univariable analysis, the cutoff value of serum UA level at 3.4 mg/dL has a significant effect on the occurrence of GVHD. The odds of GVHD incidence in patients with UA higher than 3.4 mg/L was 37% lower than patients with the UA below the cutoff ([CI: 0.44‐0.91]; *P*‐value = .15). The blood group also significantly affected the GVHD outcome. The patients with blood group A and B had 96% and 64% higher odds of incidence, consecutively compared to blood group O ([CI: 1.26‐3.2]; *P*‐value = .07), ([CI: 1.01‐2.65], *P*‐value = .23). Other factors such as diagnosed disease, CMV, and conditioning regimen did not have any significance effect on the incidence of GVHD. The results of the multivariable analysis were significant for blood group A which revealed that the odds of incidence in patients with blood group A was half the patients with blood group O ([CI: 0.28‐0.95], *P*‐value = .07).

**Table 4:** Univariable and multivariable logistic regression models for graft‐versus‐host disease

Variables	Univariable		Multivariable	
	Odds ratio (75% CI)	*P*‐value	Adjusted odds ratio (90% CI)	*P*‐value
Recipient age	0.99 (0.97‐1.00)	.56		
Diagnosed disease		.93		
NHL	1.50 (0.57‐3.93)	.62		
AML	1.03 (0.48‐2.20)	.96		
ALL	1.26 (0.57‐2.80)	.85		
Aplastic anemia	0.58 (0.17‐1.90)	.60		
Other	0.87 (0.25‐2.99)	.90		
HD (RL)	‐	‐		
CMV		.42		
Positive	1.48 (0.84‐2.60)	.42		
Negative (RL)	‐	‐		
Conditioning regimen		.53		
MAC1	1.71 (0.9‐3.26)	.33		
MAC2	1.40 (0.69‐2.82)	.58		
MAC3	2.80 (1.22‐6.42)	.15		
AA‐AF	2.80 (0.91‐8.5)	.28		
RIC (RL)	‐	‐		
Blood group		.18*		.06**
A	1.96 (1.26‐3.02)	.07*	0.51 (0.28‐0.95)	.07**
B	1.64 (1.01‐2.65)	.23*	0.62 (0.32‐1.22)	.25
AB	0.78 (0.41‐1.48)	.66	0.77 (0.30‐1.96)	.37
O(RL)	‐	‐	‐	‐
Prophylaxis regimen		.48		
CSA + MTX+ATG	1.02 (0.58‐1.81)	.95		
CSA + MTX(RL)	‐	‐		
Uric acid		.15*		.12
>3.4	0.63 (0.44‐0.91)	.15*	0.60 (0.35‐1.02)	.12
≤3.4 (RL)	‐	‐		

Abbreviation: RL, Reference level.

* Significant at 0.25.

** Significant at 0.1.

### 3.5 Association of high serum UA and other risk factors with overall survival

We applied a Cox proportional hazards model in order to determine the significant risk factors on the survival of the patients. As it is illustrated in Table 5, the hazard of death decreased in older patients by 2% ([CI: 0.96‐0.99]; *P*‐value = .20). The diagnosed disease was a risk factor which suggested a significant effect on the hazard of death (*P*‐value = .03). The hazard of death in patients with AML was almost half of those classified in subgroup “HD” ([CI: 0.23‐0.80], *P*‐value = .12).

**Table 5:** Univariable and multivariable cox regression models for overall survival

Variables	Univariable		Multivariable	
	Hazard ratio (75% CI)	*P*‐value	Adjusted hazard ratio (90% CI)	*P*‐value
Recipient age	0.98 (0.96‐0.99)	.20*	0.98 (0.96‐1.01)	.46
Diagnosed disease		.03*		.56
NHL	0.41 (0.15‐1.12)	.31	0.81 (0.64‐1.34)	.34
AML	0.43 (0.23‐0.80)	.12*	0.75 (0.41‐1.34)	.42
ALL	0.54 (0.96‐4.68)	.28	0.59 (0.81‐1.98)	.58
Aplastic anemia	2.13 (0.96‐4.68)	.38	1.78 (0.91‐2.83)	.45
Other	0.94 (0.39‐2.30)	.94	0.67 (0.88‐2.45)	.63
HD (RL)	‐	‐	‐	‐
Recipient CMV		.59		
Positive	0.79 (0.47‐1.31)	.59		
Negative(RL)	‐	‐		
Conditioning regimen		.07*		
MAC1	1.57 (0.85‐2.90)	.39	2.45 (0.45‐2.64)	.25
MAC2	2.63 (1.37‐5.02)	.08*	1.56 (0.78‐3.11)	.28
MAC3	1.07 (0.39‐2.91)	.93	3.35 ( 0.93‐1.81)	.29
AA‐AF	2.24 (0.82‐6.11)	.35	2.21 (0.99‐5.45)	.31
RIC (RL)	‐	‐	‐	‐
Blood group		.64		
A	0.96 (0.64‐1.45)	.92		
B	1.36 (0.87‐2.13)	.41		
AB	1.50 (0.91‐2.46)	.34		
O(RL)	‐	‐		
Prophylaxis regimen		.48		
CSA + MTX+ATG	2.14 (1.33‐3.41)	.06*		
CSA + MTX (RL)	‐	‐		
Uric acid		.15*		.41
>3.4	0.65 (0.47‐0.92)	.15*	0.72 (0.38‐1.38)	.41
≤3.4 (RL)	‐	‐		

Abbreviation: RL, Reference level.

* Significant at 0.25.

**Figure 1:**
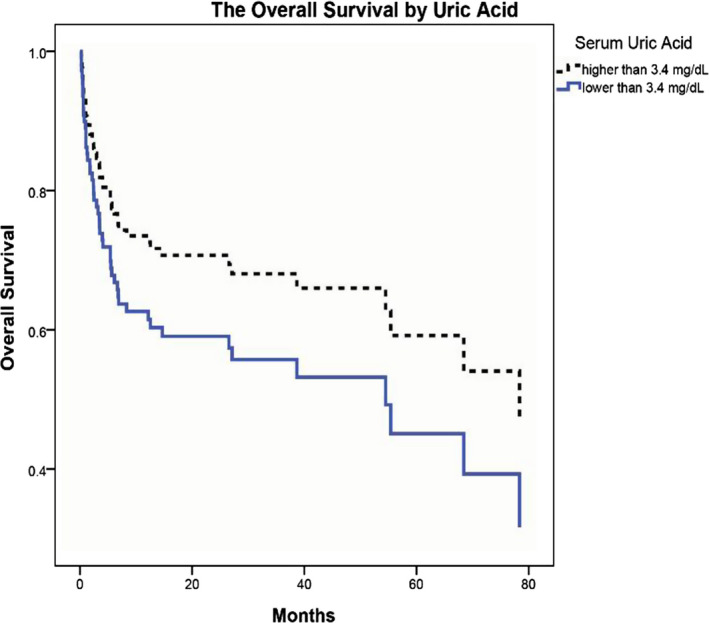
The overall survival of patients based on serum uric acid cutoff. The patients were divided based on the defined cutoff (3.4 mg/dL) into two groups. The patients with higher serum UA levels (dashed line) had better survival than those with lower serum UA levels (straight line)

The conditioning regimen was among the influential factors illustrating that in MAC2 regimen, the hazard of death was 2.63 times greater than the RIC regimen ([CI: 1.37‐5.02], *P*‐value = .08). Prophylaxis regimen of CSA + MTX+ATG increased the death hazard up to 2.14 times greater than the regimen of CSA + MTX ([CI: 1.33‐3.41], *P*‐value = .06). The patients with serum UA higher than 3.4 mg/dL had the hazard of death 35% lower than patients with UA lower than 3.4 mg/dL ([CI:0.47‐0.92], *P*‐value = .15). The survival curve illustrated that the overall survival of patients with the median serum UA levels higher than 3.4 mg/dL was better than the patients whose median UA levels were lower than 3.4 mg/dL (Figure 1). The other factors in the model were not associated with the survival of the patients. Besides, none of the variables were found to be significant in the adjusted multivariable model demonstrated in Table 5. Table 6 indicates the median of serum UA and hyperuricemia in each disease type, separately. The correlation of serum UA level with OS was also conducted. As shown, the serum UA level only in AML patients had a significant correlation with the overall survival in which the increased UA level resulted in poor survival of the patients (*P*‐value: .03).

**Table 6:** Correlation of uric acid with overall survival based on diseases

Diagnosis	Median of UA (mg/dL)	Hyperuricemia^a^ (%)	Correlation of UA with OS	
			*P*‐value	CC
NHL	2.9	30.7	.53	−0.24
HD	4	50	.08	−0.53
AML	3.3	44.8	.03*	−0.22
ALL	3.2	41.1	.06	−0.41
Aplastic anemia	2.8	16.6	.60	−0.27
Other	4.2	83	.09	−0.88

Abbreviation: CC, Coefficient of correlation.

^a^ Based on the defined cutoff (3.4 mg/dL).

* Significant at 0.05.

## 4 DISCUSSION

The radio/chemotherapy, along with the conditioning regimen before the HSCT, cause massive cell death in all organs of the patients. Dying cells release a variety of molecules which is called DAMPs or danger signals including extracellular adenosine triphosphate, UA, high mobility group box‐1 protein, etc.^12^, ^17^, ^18^ UA is of the most critical danger signals activates the innate and adaptive immunity and provokes the inflammation.^12^ Through NLRP3 inflammasome‐mediated production of IL1, UA affects the T‐cell responses, which are the most responses in GVHD. Studies have reported the role of NLRP3 inflammasome, IL1, and DAMPs in the pathogenesis of GVHD.^12^, ^19^, ^20^

The accessibility and ease of measurement together with its role in the inflammatory conditions, such as HSCT, make the serum UA as an attractive biomarker in researches to predict the GVHD.^14^ However, the role of UA in inflammation is still controversial. In allogeneic responses such as kidney transplantation, high levels of UA are associated with both cellular and humeral immune activation leading to graft rejection or poor graft survival.^21^ In HSCT, also, UA is introduced as a sensitive biomarker to distinguish the graft failure from poor graft function, but its predictive role in GVHD is a matter of debate.^15^ Preclinical and phase I clinical studies have reported that UA‐decreasing drugs, including uricase and allopurinol, can diminish the GVHD incidence and alleviate its severity.^22^, ^23^ These studies concluded that high UA level is a risk factor for GVHD and should be decreased before HSCT to control the GVHD. Albeit, these reports were about the association of UA‐decreasing drugs with GVHD.^22^, ^23^ Moreover, several studies implied the association of low UA levels and inflammatory and degenerative diseases.^24^, ^25^, ^26^

The purpose of this study was to identify the effects of serum UA on the incidence of GVHD and the overall survival with the adjusted effect of other risk factors. To achieve this goal, serum UA levels of 201 patients with hematological disorders were measured from 1 week before the allo‐HSCT until 2 weeks after transplantation. The threshold for serum UA level in three different periods, including 1 week before transplant, 1 and 2 weeks after transplant was determined using ROC curves. As it is illustrated in the results, the AUC of the cutoff determined by ROC for prediction of both GVHD incidence and overall survival in all three periods was lower than 60% which means these cutoffs have low capacity to predict the outcomes. Moreover, the sensitivity and specificity of the cutoffs were low. These results show that the thresholds achieved by ROC curve are not a reliable cutoff to predict the HSCT outcome.

The other method that is used to determine the cutoff for serum UA in our patients was median. The median of serum UA levels in the period of 1 week before the transplantation was 3.4 mg/dL, which was selected as a cutoff for the statistical analysis. Generally, the cutoff point for hyperuricemia is 6.7 mg/dL at which the crystallization of UA begins.^27^ Our determined cutoff was median and was not based on the crystallization point. The reason for choosing 1 week before HSCT to define a cutoff is that the UA levels in this period are critical in determining the fate of HSCT. Previous reports pointed out that the pre‐transplant UA decrease can affect the GVHD.^14^, ^18^, ^19^

Our univariable analysis showed that the cutoff value of serum UA level at 3.4 mg/dL has a significant effect on the occurrence of GVHD in a way that GVHD incidence in patients with UA higher than 3.4 mg/L was 37% lower than patients with the UA below the cutoff. However, the multivariable analysis showed no significant association between serum UA levels and GVHD. This finding contradicts with some previous reports which claimed that the higher serum UA is associated with GVHD incidence and its severity,^22^, ^23^ albeit it is in parallel with the report of Ostendorf et al^14^ that low UA levels in both univariable and multivariable analyses significantly associated with grade II‐IV acute GVHD. 

The probable explanation provided by Ostendorf and his colleagues for the negative association between UA levels and GVHD was the reduced antioxidative capacity in case of hypouricemia.^14^ This explanation raised from the evidence suggests that UA is the major natural antioxidant in the periphery, and reduced antioxidative capacity is associated with GVHD.^28^, ^29^, ^30^, ^31^ Except for the serum UA levels and blood group, other factors such as diagnosed disease, CMV, and conditioning regimen did not have any significant effect on the incidence of GVHD. The blood group A and B also significantly increased the GVHD incidence, compared to blood group O, which might be due to the ABO antibodies and blood group incompatibility in some patients.

In accordance with the results of GVHD, it is indicated that the lower serum UA levels are associated with a higher risk of death and inferior overall survival. It is found that patients with serum UA higher than 3.4 mg/dL had 35% lower hazard of death compared to patients with UA lower than 3.4 mg/dL. Despite the significant association of low UA levels with the GVHD incidence, Ostendorf et al^14^ reported no significant association reported between low UA levels and OS.

Of note, including the ATG to the prophylaxis regimen, increased the hazard of death up to 2.14 times greater, compared to the regimen without ATG. It should be noted that all significant results in overall survival came from univariable analysis and multivariable analysis showed no significant effect which indicates that each prognostic factor in the presence of other factors has no significant role in the prediction of OS.

Moreover, in our univariable analysis, the patients diagnosed with AML showed better OS among other diseases. Intriguingly, hyperuricemia was reported in one‐third of hematological malignancies such as AML and demonstrated to be associated with poor prognosis and poor survival in AML.^32^, ^33^, ^34^ To clarify the role of UA levels on the OS, we further illustrated the median of UA in each disease and evaluate the correlation between UA level and OS in each category. As it is reported in the result, the only disease in which the UA and OS is significantly correlated with AML which is accordant with the better OS of AML patients seen in the Cox regression model. The other diseases had no better OS and also no correlation of UA and OS. Interestingly, among the diseases, the nearest median UA level to the cutoff (3.4 mg/dL) is for AML patients and almost half of the AML patients is hyperuricemic. The correlation of UA level and OS in AML patients is negative showing higher UA level correlates with poor OS which is in line with previous studies.^32^, ^33^, ^34^ The better OS of AML patients in our study was found only in univariable model while after adjusting with other covariates including UA, this better OS is not significant anymore.

Conclusively, we demonstrated that the lower serum UA levels are associated with GVHD incidence and inferior overall survival. Our result is in line with some previous studies and contradicts some others which suggest that there might be an optimum range for UA levels which control the HSCT outcome and UA levels either lower or higher than this range could be detrimental for HSCT outcome. More comprehensive studies can evaluate the validity of this hypothesis.

## AUTHOR CONTRIBUTIONS

K.GH. SP, SS, and AH participated in designed experiments and critical revision of the manuscript; RN, MHK, and M.S performed data collection and analyzed the data. All authors revised the manuscript and approved the final paper.
